# Factors Associated with Thyroid-Related Adverse Events in Patients Receiving PD-1 or PD-L1 Inhibitors Using Machine Learning Models

**DOI:** 10.3390/cancers13215465

**Published:** 2021-10-30

**Authors:** Woorim Kim, Young-Ah Cho, Dong-Chul Kim, A-Ra Jo, Kyung-Hyun Min, Kyung-Eun Lee

**Affiliations:** 1 College of Pharmacy, Chungbuk National University, Cheongju 28160, Korea; wanppa@chungbuk.ac.kr (W.K.); minkh@chungbuk.ac.kr (K.-H.M.); 2 College of Pharmacy, Gyeongsang National University, Jinju 52828, Korea; cyapharm@gnu.ac.kr; 3 The Prime Hospital, 305 Nabulo, Jinju 52828, Korea; 4 Department of Pathology, Gyeongsang National University Hospital, Jinju 52828, Korea; kdcjes@gnu.ac.kr; 5 Department of Nursing education, Gyeongsang National University Hospital, Jinju 52828, Korea; whdkfk0912@gnuh.co.kr

**Keywords:** immune checkpoint inhibitors, risk factors, hyperthyroidism, hypothyroidism, machine learning

## Abstract

**Simple Summary:**

Although immune checkpoint inhibitors have a potential role in thyroid-related complications, no study has investigated factors associated with such adverse events. This study aims to explore the factors associated with thyroid-related adverse events in patients with anti-PD-1/PD-L1 agents by training predictive models utilizing various machine learning approaches. The results of this study could be used to develop individually tailored intervention strategies to prevent immune checkpoint inhibitor-induced thyroid-related outcomes.

**Abstract:**

Targets of immune checkpoint inhibitors (ICIs) regulate immune homeostasis and prevent autoimmunity by downregulating immune responses and by inhibiting T cell activation. Although ICIs are widely used in immunotherapy because of their good clinical efficacy, they can also induce autoimmune-related adverse events. Thyroid-related adverse events are frequently associated with anti-programmed cell death 1 (PD-1) or anti-programmed cell death-ligand 1 (PD-L1) agents. The present study aims to investigate the factors associated with thyroid dysfunction in patients receiving PD-1 or PD-L1 inhibitors and to develop various machine learning approaches to predict complications. A total of 187 patients were enrolled in this study. Logistic regression analysis was conducted to investigate the association between such factors and adverse events. Various machine learning methods were used to predict thyroid-related complications. After adjusting for covariates, we found that smoking history and hypertension increase the risk of thyroid dysfunction by approximately 3.7 and 4.1 times, respectively (95% confidence intervals (CIs) 1.338–10.496 and 1.478–11.332, *p* = 0.012 and 0.007). In contrast, patients taking opioids showed an approximately 4.0-fold lower risk of thyroid-related complications than those not taking them (95% CI 1.464–11.111, *p* = 0.007). Among the machine learning models, random forest showed the best prediction, with an area under the receiver operating characteristic of 0.770 (95% CI 0.648–0.883) and an area under the precision-recall of 0.510 (95%CI 0.357–0.666). Hence, this study utilized various machine learning models for prediction and showed that factors such as smoking history, hypertension, and opioids are associated with thyroid-related adverse events in cancer patients receiving PD-1/PD-L1 inhibitors.

## 1. Introduction

Cancer has become a global health problem and a leading cause of death worldwide. In 2020, there were approximately 19.3 million new cancer cases and 10 million cancer deaths globally. The top three cancer types in terms of the estimated number of patients are breast, lung, and prostate cancers. Lung cancer is the leading cause of cancer death [1]. Therefore, the identification of molecular mechanisms through which cancer develops and metastasizes is actively pursued; in particular, T lymphocytes, especially for antigen-directed cytotoxicity, have attracted increasing interest in developing immunotherapy for cancer treatment [2]. Various negative regulators of T cell activation act as checkpoint molecules, such as cytotoxic T lymphocyte-associated protein 4 (CTLA-4) inhibitors, anti-programmed cell death 1 (PD-1) agents, and anti-programmed cell death-ligand 1 (PD-L1) agents.

Immune checkpoint inhibitors (ICIs) have been widely used as they are highly effective against various tumors. Nivolumab, the first human IgG4 monoclonal antibody against PD-1, was approved by the FDA for various indications, including metastatic melanoma, non-small-cell lung cancer, and Hodgkin’s lymphoma [3,4,5,6]. Pembrolizumab is a human IgG4k monoclonal antibody against PD-1 that received first approval through an accelerated process as it showed a response rate of 24% in metastatic melanoma patients [7]. This agent is also approved for many other indications and has been shown to be superior to chemotherapy regimens [8]. Atezolizumab is the first PD-L1-targeted humanized IgG4 monoclonal antibody that was initially approved for the treatment of urothelial carcinoma [9]. Its usage is currently indicated for various cancers such as non-small-cell lung cancer and breast cancer [10,11].

Despite the clinical advantages of ICIs, they can induce autoimmune-related adverse events. As ICIs can activate T cells, they can give rise to various immune-related adverse events affecting various organs. Among them, thyroid-related complications have become one of the most common adverse events associated with ICIs. A meta-analysis showed that, among the patients receiving PD-1 inhibitors, 3.2% developed hyperthyroidism and 7.0% developed hypothyroidism [12]. Another case report showed that a patient with metastatic mucosal melanoma treated with ipilimumab and nivolumab developed several immune-related complications, including hypothyroidism [13]. Among the three types of ICIs (PD-1, PD-L1, and CTLA4), PD-1/PD-L1 are known to be associated with thyroid dysfunction [12,14], more frequently than CTLA4. Moreover, the incidence of thyroid-related adverse events was not affected by tumor types and ICIs used [12]. This result was also confirmed by a systematic review that showed that, regardless of the type of cancer and ICI drug used, the overall survival was similar as there is no association between the tumor type and the incidence of thyroid-related complications in patients receiving ICIs [15]. In addition, Maughan et al. showed that the frequency of most immune-related adverse events with ICIs appears to be similar across tumor types [16]. Although ICIs possibly have a role in thyroid-related complications, the factors associated with such adverse events have not been investigated yet.

As of late, machine learning methods have been increasingly used for making clinical predictions. Machine learning approaches are more suitable for developing novel prediction models than traditional statistical models that utilize variables for calculation. This study explores the factors associated with the development of thyroid-related adverse events in patients administered anti-PD-1/PD-L1 agents using training predictive models through various machine learning approaches.

## 2. Methods

### 2.1. Study Patients and Data Collection

This retrospective follow-up study included 209 patients who were prescribed ICIs between July 2015 and February 2021. Patients who had been diagnosed with hypo- or hyperthyroidism and were already prescribed thyroid-related medications or had incomplete data were excluded. Baseline values of the patient characteristics were obtained on the initial prescription date of ICIs. Data were collected using electronic medical records. Data on sex, age, height, weight, smoking history, alcohol history, comorbidities, concurrent medication, cancer type, cancer stage, and Eastern Cooperative Oncology Group performance scale (ECOGPS) were collected. Thyroid-related adverse events were defined as grade 2 or higher according to the Common Terminology Criteria for Adverse Events (CTCAE), version 5.0 [17]. The CTCAE defines grade 2 hyperthyroidism as symptomatic, thyroid suppression therapy indicated, and limiting instrumental activities of daily living. It defines hypothyroidism as symptomatic, thyroid replacement indicated, and limiting instrumental activities of daily living.

This study was approved by the Institutional Review Board of the Gyeongsang National University Hospital (approval number: GNUH 2019-11-041). All procedures performed in studies involving human participants were in accordance with the Declaration of Helsinki.

### 2.2. Statistical Analysis and Machine Learning Methods

Chi-square test or Fisher’s exact test was used to compare categorical variables between patients with thyroid-related complications and those without complications. Multivariable logistic regression analysis was used to examine independent risk factors for thyroid-related complications. Factors having a *p*-value less than 0.05 in univariate analysis along with clinically relevant confounders (age, sex, and body mass index (BMI)) were included in multivariable analysis. Odds ratios and adjusted odds ratios were calculated through univariate and multivariable analyses, respectively. To test the model’s goodness of fit, we performed a Hosmer–Lemeshow test.

This study used a random forest-based classification approach to analyze the importance of different variables for factors that are associated with thyroid-related adverse events. We focused on clinically relevant predictors and included 52 variables in the machine learning model. Seven features that are most important and clinically relevant were selected to prevent over-fitting. Machine learning methods including multivariate logistic regression, elastic net, random forest, and support vector machine (SVM) were employed for the prediction of factors affecting thyroid-related complications. All of the methods were implemented with the caret R package. To assess the ability of the associated factor to predict complication, the area under the receiver-operating curve (AUROC), the area under the precision-recall curve (AUPRC), and its 95% confidence interval (CI) of each machine learning prediction model were stated in this study. A *p*-value of less than 0.05 was considered statistically significant. A univariate statistical analysis was conducted using IBM SPSS statistics, version 20 software (International Business Machines Corp., New York, NY, USA). All other analyses were performed using R software version 3.6.0 (R Foundation for Statistical Computing, Vienna, Austria).

Internal validation was performed to measure the performance of each machine learning model. The whole dataset was randomly divided for model development and evaluation in the prediction process. After randomly partitioning one data sample into five subsets, one subset was selected for model validation while the remaining subsets were used to establish machine learning models. This five-fold cross-validation iteration was repeated 100 times to evaluate the prediction power of the machine learning models.

## 3. Results

Among the patients enrolled in this study (n = 209), 22 patients were excluded due to comorbidities of hyper- or hypothyroidism, prescription of thyroid-related medications, or incomplete data. Consequently, data on 187 patients who received ICIs were used for the analysis. The median age of the included patients was 67 years (range, 37–88 years), and there were 40 (19.1%) females. Among the ICIs, pembrolizumab was utilized the most (38.0%), followed by nivolumab (31.6%) and atezolizumab (30.5%). Twenty-three patients (12.3%) experienced thyroid-related adverse events after taking ICIs. Among them, 13, 1, and 9 patients experienced hypothyroidism, hyperthyroidism, and both, respectively.

As shown in Table 1, patients with a smoking history had more thyroid-related complications than those who did not have the history (*p* = 0.025). Additionally, patients with hypertension and heart disease had more adverse effects than those who did not have these comorbidities (*p* = 0.013 and *p* = 0.044, respectively). Patients taking P2Y_12_ inhibitors revealed more associations with thyroid-related complications compared with those without medications (*p* = 0.032) while opioids showed less complications than those without medications (*p* = 0.038).

The multivariable analysis (Table 2) included sex, age, BMI, and factors with *p* < 0.05 in univariate analysis (heart disease, P2Y_12_ inhibitors, smoking history, hypertension, and opioids). After adjusting for related covariates, patients with smoking history and hypertension showed approximately 3.7- and 4.1-fold higher incidence of thyroid-related adverse events than patients without smoking history and hypertension, respectively. Patients taking opioids revealed about 4.0-fold fewer thyroid-related complications compared with those not taking opioids. The Hosmer–Lemeshow test showed that the fitness of the multivariable analysis model was satisfactory (χ^2^ = 0.764, 4 degrees of freedom, *p* = 0.943).

**Figure 1:**
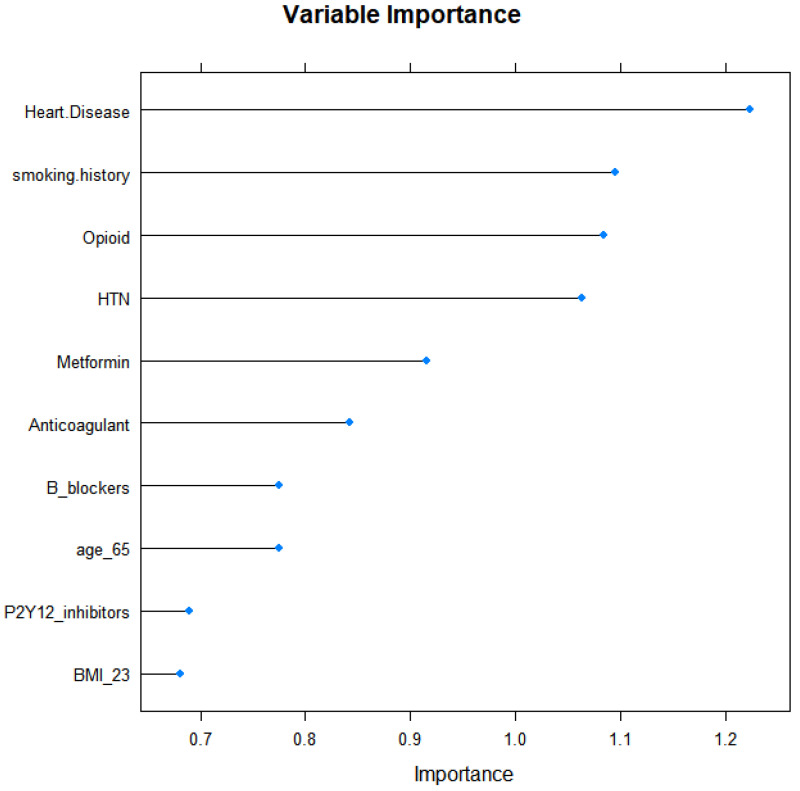
Top 10 variables by importance estimated using random forest to predict thyroid-related adverse events in patients with cancer receiving ICIs.

**Figure 2:**
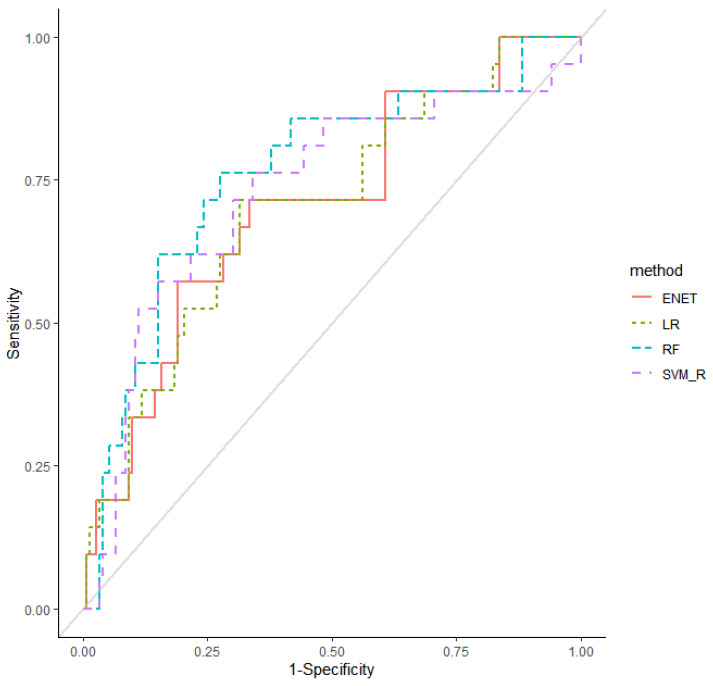
Receiver operating characteristic curves for predictive performance of the elastic net (ENET), logistic regression (LR), random forest (RF), and support vector machine radial (SVM_R) models.

As shown in Figure 1, after feature selection by performing a five-fold cross-validated random forest approach, seven important variables (heart disease, smoking history, opioids, hypertension, sex, age, and BMI) were included in machine learning models. The average AUROC values and AUPRC values after performing five-fold cross-validated multivariate logistic regression, elastic net, random forest, and SVM models across 100 random iterations are shown in Table 3. The AUROC values for multivariate logistic regression, elastic net, and random forest indicated acceptable performances of the models: 0.71, 0.71, and 0.77, respectively (95% CI 0.587–0.827, 0.588–0.829, and 0.648–0.883, respectively). Radial kernel SVM revealed sub-optimal performances of the models and an AUROC value of 0.69 (95% CI 0.539–0.838). The AUPRC values of multivariate logistic regression, elastic net, random forest, linear kernel SVM, and radial kernel SVM were 0.47, 0.47, 0.51, 0.36, and 0.45, respectively (95% CI 0.312–0.622, 0.314–0.625, 0.357–0.666, 0.216–0.497, and 0.310–0.600, respectively). For the random forest model, which showed the best prediction, the cut-off point that maximizes the accuracy was 0.05. While the prevalence of thyroid-related adverse events was 0.12, the prediction model showed an approximately 3.5-fold higher positive predictive value. Figure 2 showed the AUROC curves of the four models that exhibited acceptable or sub-optimal interpretability and prediction rates. The details for the parameters used for training models are provided in Table 4.

## 4. Discussion

The main finding of this study is that smoking history, hypertension, and opioids were associated with thyroid-related adverse events in patients taking anti-PD-1 or PD-L1. Patients with a smoking history and hypertension had an approximately 4.0-fold increased risk of thyroid-related complications compared with those without these conditions. Patients taking opioids showed an approximately 4.0-fold decreased risk of thyroid-related adverse events compared with those not taking them. Random forest was proven to be the most favorable method for predicting thyroid-related complications, with an AUROC value of 0.77 (95% CI 0.648–0.883) and an AUPRC value of 0.510 (95% CI 0.357–0.666).

Tumor antigens are known to be presented to T cells by antigen-presenting-cells, which trigger the interaction between T cell receptors and the major histocompatibility complex. Several receptors act as negative regulators of the immune response at different molecular checkpoints. For instance, the PD-1/PD-L1 pathway regulates inflammatory responses by effector T cells. Once T cells are activated, they upregulate PD-1 and inflammatory signals in the tissue. This action further induces the expression of PD-L1, resulting in the downregulation of T cell activity and protecting tissues from destruction [2]. ICIs block the PD-1/PD-L1 pathway and increase T cell activation and proliferation, which causes both anti-tumor activity and immune-related complications.

Thyroid-related adverse events are one of the most common immune-related complications in patients taking ICIs. These adverse events can present as hyperthyroidism or hypothyroidism. A randomized controlled phase 3 study showed the occurrence of both hypothyroidism (10.1% in the 2-week group and 8.7% in the 3-week group) and hyperthyroidism (6.5% and 3.2%, respectively) in patients receiving pembrolizumab [18]. Another randomized controlled trial also showed hypothyroidism and hyperthyroidism to be the most common adverse events of pembrolizumab [19]. In the group administered 2 mg/kg of pembrolizumab, 8% of the patients developed hypothyroidism while 4% developed hyperthyroidism [19]. In addition, a randomized, open-label, phase 3 trial reported several endocrine complications, including thyroid dysfunction, caused by nivolumab [20]. They showed that approximately more than 11% of patients receiving nivolumab had endocrine adverse events, most of which were observed during the initial seven months of the treatment [20]. As patients with ICI-induced thyroid dysfunction did not show clinical symptoms, it becomes crucial to carefully detect any adverse event during hormone monitoring. Therefore, immune-mediated adverse events, especially thyroid-related complications, play important roles in safety when facing the management of ICIs.

Our study results revealed that current or ex-smokers receiving anti-PD-1/PD-L1 therapy are at a higher risk of thyroid dysfunction. Cigarette smoking is a known risk factor for thyroid-related complications. A previous study has shown an association between smoking and the development of Graves’ hyperthyroidism [21]. Fukata et al. revealed that smoking increased the risk of subsequent hypothyroidism, possibly because of the antithyroid effect of thiocyanate [22]. Meanwhile, smoking is known to increase the efficacy of ICIs. A meta-analysis showed that both monotherapy and combination therapy are superior to chemotherapy in smokers; however, they were less effective than chemotherapy in never-smokers [23]. A possible explanation of this phenomenon is PD-L1 upregulation caused by smoking [24,25]. It has been shown that elevated levels of PD-L1 expression increased the efficiency of anti-PD-1/PD-L1 treatment [26,27]. As a result, increased activities of ICIs may trigger thyroid abnormalities because of the high T cell activity.

This study showed that hypertension is a risk factor for thyroid-related adverse events. The renin–angiotensin system plays a vital role in the regulation of hypertension. Increased renin–angiotensin system activity is known to increase blood pressure and to induce immunosuppression in the tumor environment [28]. Coelho et al. showed that oncogenic renin–angiotensin system signaling can increase PD-L1 expression [28]. As shown in the smoking case, it can be speculated that the anti-PD-1/PD-L1 treatment may have a higher efficacy because of upregulation.

This study showed that opioid use was negatively associated with thyroid-related complications in patients treated with PD-1/PD-L1 inhibitors. Opioids play a crucial role in increasing the resistance to immunotherapy [29]. Morphine and buprenorphine reduce the levels of interleukin-4 mRNA and protein in T cells [30]. A retrospective study on 102 cancer patients administered opioids and ICIs showed poor outcomes [31], possibly because of the presence of opioid receptors on immune cells [29]. As opioid receptors are expressed in immune cells, opioids could alter immune responses [32]. Given that the use of opioids can dysregulate the immune response, opioid usage during ICI treatment could affect its efficacy and can cause autoimmune complications, including thyroid dysfunction.

The utilization of machine learning approaches to predict thyroid-related adverse events in patients receiving PD-1/PD-L1 inhibitors is a novel concept in clinical research. Machine learning algorithms are integrated into the clinical decision-making process to guide clinicians to diagnose, screen, prevent, and treat cancer patients. Machine learning methods use a training dataset to train computational models and to generate the most optimal prediction models, which can further be validated in the test dataset to ensure accuracy. In clinical settings, these models can help predict and manage thyroid-related complications in patients receiving ICIs. In a binary model such as this study, the outcome prediction performance of a model is evaluated by the ROC curve. In this study, we performed feature selection using random forest, an ensemble method of bootstrap aggregated binary classification trees [33], to overcome overfitting. We also trained various machine learning models and concluded that the random forest model outperformed the other models with the highest AUROC and AUPRC values. Hence, this model can be used for predicting thyroid-related complications in patients on ICIs.

The limitations of our study are its small sample size and the lack of a detailed mechanism. Although it has been reported that cancer types did not affect thyroid-related adverse events, 10 types of cancer and 3 ICIs could influence other clinical outcomes, thereby complicating the study outcome. Therefore, cautious interpretation is needed when applying the results of this study to real clinical settings. Moreover, because of the lack of independent data, we did not perform the external validation that is needed to examine the trained model’s performance. Further research is needed to externally validate current results to ensure accuracy for application in clinical settings. Nevertheless, to the best of our knowledge, this is the first study to investigate factors responsible for thyroid dysfunction in patients taking anti-PD-1/PD-L1 agents. In addition, this study provides important features and prediction models based on machine learning algorithms, which included logistic regression, elastic net, random forest, and SVM. Given that our study developed prediction models using the factors associated with thyroid-related adverse events in patients receiving ICIs, our findings provide additional insight to manage thyroid-related complications. Moreover, the results of this study could be utilized to design and develop individually tailored PD-1/PD-L1 inhibitor treatments for various cancer types.

## Figures and Tables

**Table 1:** Factors associated with thyroid-related adverse events in patients receiving immune checkpoint inhibitors.

Characteristics		Complication (n = 23)	No Complication (n = 164)	*p*-Value
Sex				1.000
	Male	18 (78.3)	129 (78.7)	
	Female	5 (21.7)	35 (21.3)	
Age				0.420
	<65	11 (47.8)	64 (39)	
	≥65	12 (52.2)	100 (61)	
BMI				0.921
	<23	13 (61.9)	93 (60.8)	
	≥23	8 (38.1)	60 (39.2)	
Smoking history				0.025
	Yes	9 (39.1)	29 (17.7)	
	No	14 (60.9)	135 (82.3)	
Alcohol history				0.115
	Yes	4 (17.4)	12 (7.3)	
	No	19 (82.6)	152 (92.7)	
Comorbidities				
Hypertension				0.013
	Yes	14 (60.9)	56 (34.1)	
	No	9 (39.1)	108 (65.9)	
Hyperlipidemia				0.052
	Yes	5 (21.7)	13 (7.9)	
	No	18 (78.3)	151 (92.1)	
COPD				0.477
	Yes	1 (4.3)	18 (11)	
	No	22 (95.7)	146 (89)	
Diabetes mellitus				0.689
	Yes	5 (21.7)	42 (25.6)	
	No	18 (78.3)	122 (74.4)	
Gout				1.000
	Yes	0 (0)	4 (2.4)	
	No	23 (100)	160 (97.6)	
BPH				0.136
	Yes	0 (0)	19 (11.6)	
	No	23 (100)	145 (88.4)	
Parkinson’s disease				1.000
	Yes	0 (0)	1 (0.6)	
	No	23 (100)	163 (99.4)	
Osteoporosis				1.000
	Yes	0 (0)	2 (1.2)	
	No	23 (100)	162 (98.8)	
MI				0.075
	Yes	2 (8.7)	2 (1.2)	
	No	21 (91.3)	162 (98.8)	
Heart disease				0.044
	Yes	4 (17.4)	8 (4.9)	
	No	19 (82.6)	156 (95.1)	
Asthma				1.000
	Yes	0 (0)	3 (1.8)	
	No	23 (100)	161 (98.2)	
Buger’s disease				1.000
	Yes	0 (0)	1 (0.6)	
	No	23 (100)	163 (99.4)	
Angina				0.123
	Yes	1 (4.3)	0 (0)	
	No	22 (95.7)	164 (100)	
Crohn’s disease				1.000
	Yes	0 (0)	1 (0.6)	
	No	23 (100)	163 (99.4)	
HIV				1.000
	Yes	0 (0)	2 (1.2)	
	No	23 (100)	162 (98.8)	
Hepatitis B				1.000
	Yes	0 (0)	3 (1.8)	
	No	23 (100)	161 (98.2)	
Concomitant drug				
Statins				0.771
	Yes	3 (13)	29 (17.7)	
	No	20 (87)	135 (82.3)	
PPIs				0.952
	Yes	8 (34.8)	56 (34.1)	
	No	15 (65.2)	108 (65.9)	
5-HT₃ Antagonists				0.625
	Yes	3 (13)	16 (9.8)	
	No	20 (87)	148 (90.2)	
D2 antagonists				0.231
	Yes	1 (4.3)	1 (0.6)	
	No	22 (95.7)	163 (99.4)	
Corticosteroids				1.000
	Yes	1 (4.3)	7 (4.3)	
	No	22 (95.7)	157 (95.7)	
Antihistamines				0.204
	Yes	3 (13)	10 (6.1)	
	No	20 (87)	154 (93.9)	
Diuretics				1.000
	Yes	1 (4.3)	12 (7.3)	
	No	22 (95.7)	152 (92.7)	
β-blockers				0.061
	Yes	3 (13)	5 (3)	
	No	20 (87)	159 (97)	
P2Y_12_ inhibitors				0.032
	Yes	4 (17.4)	7 (4.3)	
	No	19 (82.6)	157 (95.7)	
5HT₄ agonists				0.327
	No	22 (95.7)	162 (98.8)	
	Yes	1 (4.3)	2 (1.2)	
Antiepileptics				1.000
	Yes	0 (0)	1 (0.6)	
	No	23 (100)	163 (99.4)	
Antibiotics				0.744
	Yes	2 (8.7)	21 (12.8)	
	No	21 (91.3)	143 (87.2)	
Alpha-blockers				0.476
	Yes	1 (4.3)	19 (11.6)	
	No	22 (95.7)	145 (88.4)	
5α-Reductase inhibitors				1.000
	Yes	1 (4.3)	12 (7.3)	
	No	22 (95.7)	152 (92.7)	
NSAIDs				0.261
	Yes	2 (8.7)	32 (19.5)	
	No	21 (91.3)	132 (80.5)	
Metformin				0.185
	Yes	5 (21.7)	19 (11.6)	
	No	18 (78.3)	145 (88.4)	
Antipsychotics				0.327
	Yes	1 (4.3)	2 (1.2)	
	No	22 (95.7)	162 (98.8)	
Anticoagulants				0.350
	Yes	5 (21.7)	23 (14)	
	No	18 (78.3)	141 (86)	
ACE inhibitors/ARBs				0.684
	Yes	2 (8.7)	12 (7.3)	
	No	21 (91.3)	152 (92.7)	
Zolpidem				0.327
	Yes	1 (4.3)	2 (1.2)	
	No	22 (95.7)	162 (98.8)	
TCAs				1.000
	Yes	0 (0)	1 (0.6)	
	No	23 (100)	163 (99.4)	
Opioids				0.038
	Yes	12 (52.2)	120 (73.2)	
	No	11 (47.8)	44 (26.8)	
Aspirin				1.000
	Yes	0 (0)	5 (3)	
	No	23 (100)	159 (97)	
Dopamine				0.327
	Yes	1 (4.3)	2 (1.2)	
	No	22 (95.7)	162 (98.8)	
Benzodiazepines				0.738
	Yes	3 (13)	19 (11.6)	
	No	20 (87)	145 (88.4)	
Antivirals				1.000
	Yes	0 (0)	3 (1.8)	
	No	23 (100)	161 (98.2)	
SSRIs, SNRIs				0.600
	Yes	0 (0)	7 (4.3)	
	No	23 (100)	157 (95.7)	
Cancer stage				0.428
	1	0 (0.0)	1 (0.6)	
	2	0 (0.0)	3 (1.8)	
	3	3 (13.0)	9 (5.5)	
	4	20 (87.0)	150 (92.0)	
Diagnosis				0.223
	Bladder cancer	0 (0)	15 (9.1)	
	Colon cancer	0 (0)	3 (1.8)	
	Gastric cancer	0 (0)	8 (4.9)	
	Hepatocellular cancer	1 (4.3)	12 (7.3)	
	Lung cancer	19 (82.6)	75 (45.7)	
	Pancreatic cancer	0 (0)	2 (1.2)	
	Rectal cancer	0 (0)	3 (1.8)	
	Renal cancer	0 (0)	3 (1.8)	
	Stomach cancer	0 (0)	3 (1.8)	
	Other	2 (8.7)	38 (23.2)	
ECOGPS				0.464
	0	0 (0)	1 (0.6)	
	1	21 (91.3)	129 (79.6)	
	2	2 (8.7)	18 (11.1)	
	3	0 (0)	14 (8.6)	

BMI: body mass index: COPD: chronic obstructive pulmonary disease; BPH: benign prostatic hyperplasia; MI: myocardial infarction; HIV: human immunodeficiency viruses; PPIs: proton pump inhibitors; NSAIDs: non-steroidal anti-inflammatory drugs; ACE: angiotensin-converting enzyme; ARBs: angiotensin receptor blockers; TCAs: tricyclic antidepressants; SSRIs: selective serotonin reuptake inhibitors; SNRIs: serotonin and norepinephrine reuptake inhibitors; ECOGPS: Eastern Cooperative Oncology Group performance status.

**Table 2:** Multivariable analysis to identify predictors for thyroid-related adverse events in patients receiving immune checkpoint inhibitors.

Characteristics	Crude OR (95% CI)	*p*-Value	Adjusted OR (95% CI)	*p*-Value
Sex	1.024 (0.355–2.952)	0.965		
Age < 65	0.698 (0.291–1.677)	0.422		
BMI	0.954 (0.373–2.438)	0.921		
Heart disease	4.105 (1.129–14.932)	0.032		
P2Y_12_ inhibitors	4.722 (1.265–17.631)	0.021		
Smoking history	2.993 (1.183–7.574)	0.021	3.748 (1.338–10.496)	0.012
Hypertension	3.000 (1.223–7.360)	0.016	4.093 (1.478–11.332)	0.007
Opioids	0.400 (0.165–0.972)	0.043	0.248 (0.090–0.683)	0.007

Crude OR: the result from fitting the univariate logistic regression model. Adjusted OR: adjusted for sex, age, BMI, heart disease, P2Y_12_ inhibitors, smoking history, hypertension, and opioids. BMI: body mass index; OR: odds ratio; CI: confidence interval.

**Table 3:** Comparisons of AUC for the logistic regression, elastic net, random forest, and SVM models.

Machine Learning Model	AUROC (95% CI)	AUPRC (95% CI)
Logistic regression	0.71 (0.587–0.827)	0.47 (0.312–0.622)
Elastic net	0.71 (0.588–0.829)	0.47 (0.314–0.625)
Random Forest	0.77 (0.648–0.883)	0.51 (0.357–0.666)
SVM (Linear)	0.57 (0.394–0.752)	0.36 (0.216–0.497)
SVM (Radial)	0.69 (0.539–0.838)	0.45 (0.310–0.600)

AUROC: area under the receiver-operating curve; AUPR: area under the precision-recall curve; CI: confidence interval; and SVM: support vector machine.

**Table 4:** Machine learning model specifics.

Method	Hyperparameter	
	Model Specification and Search Grids	Selected Values
Elastic net	λ: 100 equally spaced values in logarithmic scale between 10^−4^ and 0	λ: 0.01261857
	α: 0, 0.2, 0.4, 0.6, 0.8, 1	α: 0.6
Random forests	mtry: 1, 2, 3, 4, 5, 6, 7	mtry: 1
SVM with linear kernel	C: 0, 0.001, 0.005, 0.01, 0.05, 0.1, 0.25, 0.5, 0.75, 1, 1.25, 1.5, 1.75, 2, 5	C: 1
SVM with radial kernel	Sigma: 2^−15^, 2^−13^, 2^−11^, 2^−9^, 2^−7^, 2^−5^, 2^−3^, 2^−1^, 2, 2^3^	Sigma: 0.125
	C: 2^−5^, 2^−3^, 2^−1^, 2, 2^3^, 2^5^, 2^7^, 2^9^, 2^11^, 2^13^, 2^15^	C: 128

SVM: support vector machine.

## Data Availability

The data presented in this study are available from the corresponding author upon request.
